# Evolutionary sequence analysis of complete eukaryote genomes

**DOI:** 10.1186/1471-2105-6-53

**Published:** 2005-03-11

**Authors:** Jaime E Blair, Prachi Shah, S Blair Hedges

**Affiliations:** 1 NASA Astrobiology Institute and Department of Biology, The Pennsylvania State University, 208 Mueller Laboratory, University Park, Pennsylvania 16802-5301, USA

## Abstract

**Background:**

Gene duplication and gene loss during the evolution of eukaryotes have hindered attempts to estimate phylogenies and divergence times of species. Although current methods that identify clusters of orthologous genes in complete genomes have helped to investigate gene function and gene content, they have not been optimized for evolutionary sequence analyses requiring strict orthology and complete gene matrices. Here we adopt a relatively simple and fast genome comparison approach designed to assemble orthologs for evolutionary analysis. Our approach identifies single-copy genes representing only species divergences (panorthologs) in order to minimize potential errors caused by gene duplication. We apply this approach to complete sets of proteins from published eukaryote genomes specifically for phylogeny and time estimation.

**Results:**

Despite the conservative criterion used, 753 panorthologs (proteins) were identified for evolutionary analysis with four genomes, resulting in a single alignment of 287,000 amino acids. With this data set, we estimate that the divergence between deuterostomes and arthropods took place in the Precambrian, approximately 400 million years before the first appearance of animals in the fossil record. Additional analyses were performed with seven, 12, and 15 eukaryote genomes resulting in similar divergence time estimates and phylogenies.

**Conclusion:**

Our results with available eukaryote genomes agree with previous results using conventional methods of sequence data assembly from genomes. They show that large sequence data sets can be generated relatively quickly and efficiently for evolutionary analyses of complete genomes.

## Background

The use of complete genomes for phylogenetic analysis has greatly improved our understanding of prokaryote evolution [1-3]. However, until recently, relatively few complete genome sequences were available for such analyses in eukaryotes. As this improves, there will be a greater demand on methodology for evolutionary analysis of complete genomes. Previous whole-genome studies of eukaryotes have focused on gene and gene family presence-absence [4-7], lineage-specific gene loss [8,9], insertion-deletion markers and introns [6,10,11], and other non-sequence based information. While these approaches have their advantages, previous studies have not used complete genome sequences (nucleotides and/or amino acids) for reconstructing evolutionary relationships. At the same time, the complexity of eukaryote genomes, with numerous gene duplications and losses in different lineages, has created a challenge for sequence-based phylogeny estimation. Here, we outline a conservative approach designed to utilize the wealth of evolutionary information present in complete genome sequences by identifying orthologs in multiple eukaryotes for the purpose of evolutionary analysis.

**Figure 1:**
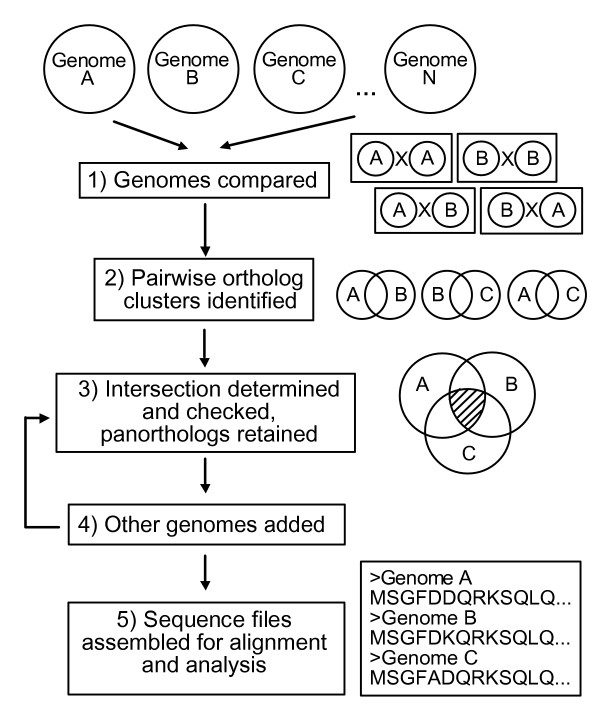
Flowchart of multigenome intersection approach (MIA). 1) Complete genomes are reciprocally compared against themselves and all other genomes with BLAST. 2) Pairwise ortholog clusters are identified using similarity scores and imported into a local database. 3) The intersection between genomes is determined by iteratively comparing sequence identification tags and retaining those clusters showing panorthology. 4) Additional genomes are added and checked as in the previous step. 5) Sequence data files are generated for evolutionary analysis.

Methods for the identification of clusters of orthologs and lineage-specific paralogs have proven useful for classifying gene function and identifying cases where genes have been differentially lost or duplicated in different lineages [12-14]. However, such assemblages of data contain a mixture of orthologs, paralogs, and missing data as a result of gene loss, and are not generally suitable for large-scale phylogenetic sequence analysis of organismal evolution. Our approach for comparing multiple genome sequences involves the identification of single-copy orthologs across a number of genomes for evolutionary analysis (Figure 1). We refer to such strict (1:1) orthologs as *panorthologs*, in reference to their presumed "complete" orthology, in contrast to *synorthologs*, which contain a mixture of species divergences and gene duplication events. In other words, panorthologs are those genes (or clusters of sequences) that contain only species divergences and do not contain in-paralogs, out-paralogs, or co-orthologs [15]. On the other hand, synorthologs are those genes (or clusters of sequences) that contain species divergences and any combination of paralogy (in-paralogs and out-paralogs). While the use of panorthologs is conservative and reduces the number of usable genes or proteins, it also lowers the probability that errors will be made in confusing a species divergence with a gene duplication event. Because the ability to identify orthologs is diminished in analyses of small to moderate numbers of species or genomes, such a conservative method is appropriate in those cases. This conservative approach has been used to identify the number of shared, unduplicated proteins in *Homo sapiens*, *Drosophila melanogaster*, *Caenorhabditis elegans*, and *Saccharomyces cerevisiae*, where it was determined that such proteins perform primarily anabolic rather than catabolic functions [16].

We compare our phylogenetic results and divergence time estimates for an analysis of seventeen published eukaryote genomes to a previous study that assembled nuclear protein sequence data in a more conventional manner from public databases [17]. While the phylogenetic relationships between the organisms included in this study are not controversial, with the exception of the position of nematodes [18], this general approach will prove useful as more genomes, including those with questionable phylogenetic affinity, are sequenced. In addition, this approach facilitates the estimation of divergence times between organisms with numerous molecular clock methods.

## Results

The number of orthologous clusters per pairwise comparison and the percentage of those clusters showing panorthology are presented in Table 1. On average, pairwise orthologous clusters contained approximately 60.3% panorthologs; exceptions include comparisons between fungi, including *Encephalitozoon *(average 89% panorthology), and all comparisons with *Arabidopsis *(average 34.6% panorthology). Comparisons within metazoans averaged 54.7% panorthology, with *Mus *and *Rattus *showing the highest number of shared transcripts (16,413 orthologous clusters; 79.2% panorthology) as expected due to their recent evolutionary divergence. Previous analyses showed approximately 12,400 panorthologs between *Mus *and *Rattus *[19]. *Caenorhabditis elegans *and *C. briggsae*, who diverged roughly 100 Ma [20], also shared a large number of transcripts (12,416 orthologous clusters; 84.4% panorthology), which is similar to a previous estimate of 12,155 panorthologs [21]. The number of orthologous clusters between *Drosophila *and *Anopheles *found here (7072, 61.3% panorthology) is also similar to a previous estimate of approximately 6130 panorthologs [22]. Pairwise comparisons with the smallest genome, the *Guillardia *nucleomorph, averaged ~176 orthologous clusters, but the percentage of panorthologs varied greatly, from a low of 25.1% with *Arabidopsis *to as high as 97.2% with *Encephalitozoon*.

**Figure 2:**
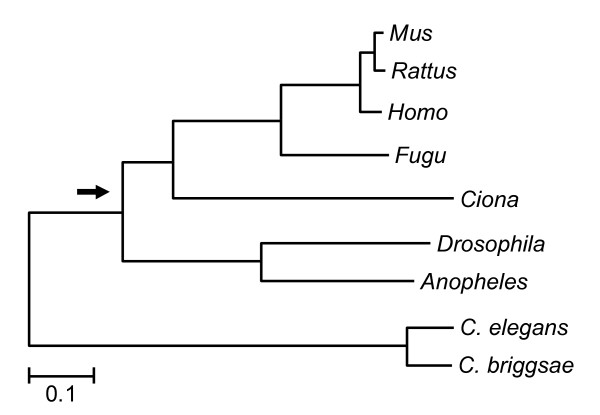
Neighbor-joining tree of nine metazoan genomes, 285 panorthologs (97,581 amino acid positions, alpha = 1.28). All nodes are supported significantly (>95%) in bootstrap analyses of neighbor-joining and maximum likelihood. The arrow indicates an alternative root [6, 18].

**Figure 3:**
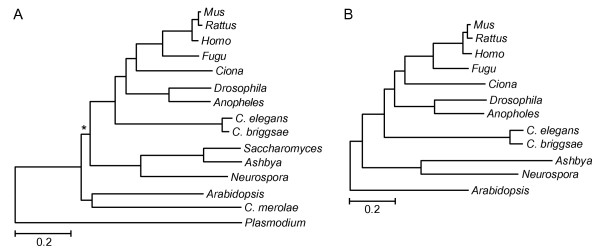
Neighbor-joining trees of complete eukaryotic genome sequence analyses. (A) The intersection of fifteen eukaryotic genomes, 10 panorthologs (5094 amino acid positions, alpha = 1.01). (B) The intersection of genomes from twelve multicellular eukaryotes, 63 panorthologs (23,571 amino acid positions, alpha = 1.15). All nodes are supported significantly (>95%) in bootstrap analyses of neighbor-joining and maximum likelihood, with the exception of node indicated by an asterisk (94% with maximum likelihood) in (A).

The intersection of nine metazoan genomes resulted in a large number of shared genes. Among the nine genomes, 285 panorthologs were found, totaling 97,581 amino acids. The neighbor-joining tree of that concatenation is shown in Figure 2; all nodes in this tree received 100% bootstrap support. The intersection of all seventeen eukaryote genomes included in this study resulted in three shared genes (t-complex protein delta subunit, proteasome beta type-1 subunit, and Nip7p biogenesis factor) and orthology was confirmed manually. The reconstructed trees for the three genes showed long-branch attraction errors associated with the intracellular parasite *Encephalitozoon *and the *Guillardia *nucleomorph (data not shown). This was expected because both have highly reduced genomes and high rates of substitution across many genes as a result of their current or ancestral parasitic and symbiotic lifestyles [23,24]. For this reason, the intersection of the remaining fifteen genomes was determined, resulting in ten panorthologs. The intersection of genomes from twelve multicellular eukaryotes resulted in 63 panorthologs. The functional classifications of the panorthologs found here are similar to those identified in previous studies [14], with the most frequently represented functions being transcription, translation, replication and repair, and RNA processing. The phylogenetic trees reconstructed from the concatenated datasets both showed the expected relationships (Figure 3a and 3b) [17]. All nodes in these trees received very high bootstrap support, with only one node showing less than 95% bootstrap support (animals + fungi in Figure 3a). The long branch observed in *Plasmodium *(Figure 3a) may be the result of both the long evolutionary separation from the other eukaryotes included in this study, and the high (A-T) composition of the genome [25] leading to biased amino acid compositions among proteins [26].

Phylogenetic trees were also reconstructed for each panortholog to test for congruence with well-supported phylogenies from the concatenated data (see Additional file 1). We found that in most cases, the consensus values calculated from individual trees agree with the high bootstrap support of the concatenated analysis. Two exceptions were the low consensus values for the accepted close relationship between animals and fungi and the contested position of nematodes. Both taxa showed slightly longer branch lengths, and long-branch attraction artifacts [27] may be affecting the individual datasets, causing low consensus values. Also, recent empirical [28] and simulation [29] studies suggest that results from multigene analyses are more accurate when a tree is derived from a concatenated dataset of individual genes rather than a consensus of trees from multiple analyses.

**Figure 4:**
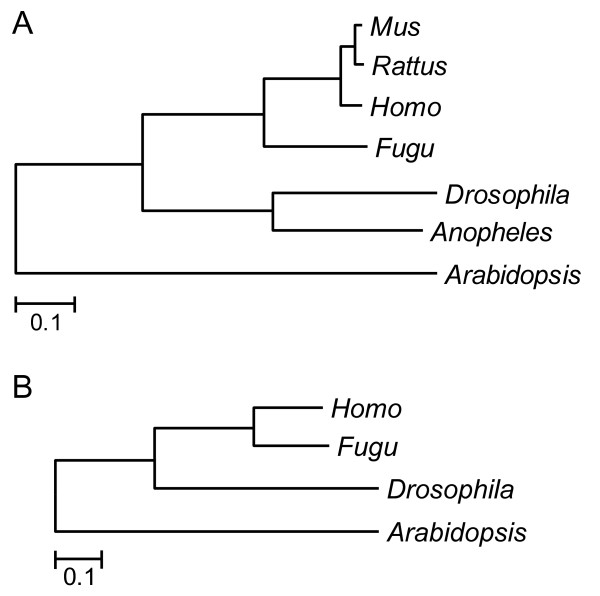
Neighbor-joining trees of genomes used to address deuterostome-arthropod divergence time. (A) The intersection of seven eukaryotic genomes, 380 panorthologs (132,190 amino acid positions, alpha = 1.38). (B) The intersection of four eukaryotic genomes, 753 panorthologs (287,000 amino acid positions, alpha = 1.46). All nodes are supported significantly (>95%) in bootstrap analyses of neighbor-joining and maximum likelihood.

Divergence times were estimated for both the 15-genome and 12-genome datasets (Table 2). Results were consistent with previous studies [17,30-33], showing an early divergence between plants, animals, and fungi (animals/fungi vs. plants ~1670 Ma, animals vs. fungi ~1400 Ma), and a Precambrian origin for animals (~900–1100 Ma). To specifically address the deuterostome-arthropod divergence within animals, two additional datasets were assembled to maximize the number of proteins analyzed: the intersection of seven genomes (*Homo*, *Mus*, *Rattus*, *Takifugu*, *Drosophila*, and *Anopheles*; *Arabidopsis *as outgroup) and the intersection of four genomes (*Homo*, *Takifugu*, and *Drosophila*; *Arabidopsis *as outgroup). The seven genome intersection contained 380 panorthologs (132,190 amino acids; Figure 4a), and yielded a vertebrate-arthropod divergence time of 955 Ma. The four genome intersection contained 753 panorthologs (287,000 amino acids; Figure 4b) and yielded a vertebrate-arthropod divergence time of ~1100 Ma. Although this last estimate was derived from more than five times the data previously used, the divergence time is remarkably consistent with previous large-scale studies [17], and suggests that bilaterian animals originated hundreds of millions of years before the first fossil evidence of their existence in the Cambrian (~520 Ma). With the exception of the maximum fossil-based time estimate used in the tetrapod-actinopterygian fish calibration, the other fossil constraints used here are minimums, and therefore the resulting time estimates are minimums [34]. The agreement between our results and those of previous studies using different methods of data assembly suggests that our genome intersection approach is correctly assembling orthologs. Younger time estimates of the vertebrate-arthropod divergence have been obtained in some studies [35-37]. However, those results are problematic because they also contain estimates which are inconsistent (too young) with undisputed aspects of the fossil record, such as the oldest red algae (1200 Ma), green algae (1000 Ma), and stramenopiles (1000 Ma) [38-40]. Possible reasons for their inconsistency are discussed elsewhere [41].

## Discussion

The complete genome sequence of an organism is essentially the maximum amount of discrete, genetically encoded information available for evolutionary analyses. However, orthology determination has been a major obstacle in the analysis of complete genomes, especially in eukaryotes where considerable gene duplication and loss has created additional complexity. Our approach for evolutionary analysis of complete eukaryote genome sequences is both simple and fast compared with the conventional method of gene-by-gene orthology determination using similarity searches in the public databases. The results of this approach applied to a subset of the available eukaryote genomes show agreement with previous results using conventional (non-genomic) approaches. In addition, the relatively high consensus values for most nodes indicate general agreement in tree topology among individual panorthologs.

The relatively low number of common genes in our intersections of 12–17 genomes is a combination of using panorthologs and including distantly related species. Genes are more likely to duplicate over long periods of evolutionary time, as in the time elapsed since plants separated from animals (~1600 Ma) [17,32]. Therefore, a better approach with such distantly related species (e.g., all eukaryotes), and those groups with high levels of gene duplication and gene loss (e.g., nematodes), would be to relax the orthology criterion and include synorthologs. In that case, a representative or consensus sequence may be chosen from among in-paralogs. On the other hand, the implementation used here (panorthologs) should yield many genes in analyses of genomes from closely related species (e.g., within mammals), even if large numbers of species are used.

The use of sequence data for comparative genomics and phylogenetics has several advantages over the use of datasets based on the presence and absence or position of genes, introns and insertions. Sequence data can provide a larger number of characters for analysis, yielding hundreds of thousands of amino acid sites and more than a million nucleotide sites in some cases. Also, statistical models of sequence change are better known than those for non-sequence based data. Finally, the assembly of sequence data from complete genomes of multiple organisms not only facilitates phylogenetic and divergence time analyses, but a diversity of other comparative evolutionary analyses requiring sequence information [42,43].

## Conclusion

Unlike previous studies of complete eukaryote genomes, here we have used a fast, conservative approach to assemble orthologous clusters of proteins for phylogenetic analysis and divergence time estimation. Our results are similar to previous studies that used conventional (slower) gene-by-gene data mining. We find that complete genome sequences support the close evolutionary relationship between animals and fungi, and also that molecular divergences between animals occurred approximately 400 million years before the Cambrian Explosion of fossils. Our approach will be further tested as more eukaryote genomes are sequenced.

## Methods

Multigenome intersection approach for evolutionary analysis (MIA): Reciprocal BLAST [44] searches of genomes versus themselves and versus all other genomes included in the analysis were used to generate lists of pairwise similarity scores for each transcript. These scores were then used to generate orthologous clusters of proteins by first determining the "primary" ortholog pair through reciprocal best hits, then adding lineage-specific paralogs (in-paralogs) as implemented in INPARANOID [45]. The settings used here (sequence overlap cut-off 50%, group overlap cut-off 50%, in-paralog confidence cut-off 5%) were considered optimal in the sense that all closely related lineage-specific paralogs (and alternative transcripts) will be placed in the same pairwise cluster, minimizing the probability that the same gene will be represented in more than one cluster. In-paralogs are added to clusters if they are more similar to one member of the primary ortholog pair than the two primary orthologs are to each other [45].

Only pairwise ortholog clusters can be obtained using INPARANOID. For phylogenetic analysis, ortholog sets for a larger number of genomes (at least four) must be constructed. Therefore, we combined the pairwise ortholog clusters from groups of species using a relational database. The intersection between ortholog clusters was determined by iteratively comparing sequence identification tags and reducing the intersected clusters at each round to exclude clusters that represent relationships other than panorthology. For example, consider genomes A, B and C. First, ortholog clusters are determined for each pairwise genome comparison, which results in clusters: A-B, B-C and A-C. Intersection of sets A-B and B-C is obtained by searching for common sequences of genome B in the two sets and merging the two sets accordingly into an ortholog cluster set of A-B-C. This combined set is reduced to include only clusters with panorthology relationships, i.e. only clusters with one sequence for each of the genomes A, B and C are retained. Further, the combined cluster A-B-C is compared with the pairwise cluster A-C based on common sequence tags for genome C. Any cluster from the combined A-B-C set that does not agree with the sequence grouping of genomes A and C is removed. This last step serves as an important check for orthology in each iteration of the intersection procedure, and is similar to the construction of three-member COGs (clusters of orthologous groups) [46]. The steps described above were performed iteratively in order to add more species to the ortholog clusters. Any number of genomes can be intersected (tested up to seventeen here), and an outgroup can be treated as part of the intersection or added separately by using a pairwise comparison to one of the in-group taxa. All programming was written in Perl.

Analysis of Eukaryote Genomes: Complete protein transcripts were obtained for the following eukaryote genomes [three letter abbreviation]: *Homo sapiens *[Hsa] (version 34b.2) [16,47,48]; *Mus musculus *[Mmu] (version 32.2) [48,49]; *Rattus norvegicus *[Rno] (version 3b.1) [19,48]; *Takifugu rubripes *[Tru] (version 3.0) [50,51]; *Ciona intestionalis *[Cin] (version 1.0) [51,52]; *Drosophila melanogaster *[Dme] (version 3.1) [53,54]; *Anopheles gambiae *[Aga] (version 2a.2) [48,55]; *Caenorhabditis elegans *[Cel] (version 120) [56,57]; *Caenorhabditis briggsae *[Cbr] (version 25) [21,57]; *Saccharomyces cerevisiae *[Sce] [58,59]; *Neurospora crassa *[Ncr] (version 3) [60,61]; *Ashbya gossypii *[Ago] (version 1.0) [62,63]; *Encephalitozoon cuniculi *[Ecu] [24,64]; *Arabidopsis thaliana *[Ath] (version 5.0) [65,66]; *Cyanidoschyzon merolae *[Cme] [67,68]; *Guillardia theta *nucleomorph [Gtn] [23,64]; and *Plasmodium falciparum *3D7 [Pfa] (version 4.1) [25,69]. Some genome transcripts were given unique sequence identifiers to avoid redundancy when sequence tags are truncated. The intersection of these seventeen genomes was determined as described above.

Each panortholog was aligned [70] and individual datasets were concatenated. Both individual panorthologs and concatenations were analyzed using maximum likelihood [71] to determine alpha parameters for the gamma rate-heterogeneity correction. Phylogenetic trees of concatenated datasets were reconstructed using neighbor-joining (Poisson + gamma correction model) with bootstrapping (2000 replicates) [72] and maximum likelihood (JTT + gamma correction model) with 1000 puzzling steps [73]. Phylogenetic trees of individual datasets were reconstructed using maximum likelihood (Poisson + gamma correction model) [71] and a consensus tree was derived [74]. Consensus values (i.e. the proportion of trees recovering a specific node) were calculated for each dataset.

Divergence times were estimated for concatenated datasets using Bayesian inference (JTT model) [75] as described previously [17]. The following fossil dates were used as minimum constraints: *Mus-Rattus *12 Ma [76], primate-rodent 65 Ma [77], tetrapod-actinopterygian fish 425 Ma (lower bound) and 495 Ma (upper bound) [78], vertebrate-urochordate 520 Ma [79], *Drosophila-Anopheles *250 Ma [80], chordate-arthropod 545 Ma [77], green algae/plants-red algae 1200 Ma [40].

## Authors' contributions

JEB and PS designed and developed the methodology. PS programmed the genome intersection software. JEB carried out the evolutionary analyses and drafted the manuscript. SBH coordinated the research and assisted with drafting the manuscript.

## Supplementary Material

Additional File 1 Consensus trees of individual gene trees. Consensus trees of individual gene (panortholog) trees, showing percentage of individual gene trees supporting each node. Click here for file

## Figures and Tables

**Table 1:** Number of orthologous clusters (upper-right) and percentage panorthologs (lower-left) per pairwise comparison^a^.

	**Hsa**	**Mmu**	**Rno**	**Tru**	**Cin**	**Dme**	**Aga**	**Cel**	**Cbr**	**Sce**	**Ncr**	**Ago**	**Ecu**	**Ath**	**Cme**	**Gtn**	**Pfa**
**Hsa**		14571	14201	9881	6100	5009	5081	3794	4114	1898	2197	1856	759	2892	1628	177	1198
**Mmu**	68.5		16413	9885	6115	4933	5112	4194	4182	1924	2240	1887	755	2894	1523	183	1241
**Rno**	73.2	79.2		9708	6016	5001	5023	4127	4079	1871	2207	1825	754	2836	1630	177	1235
**Tru**	61.6	61	67.5		4872	4970	4974	4109	4090	1464	2224	1808	725	2814	1591	177	1260
**Cin**	49.3	50.3	55.6	58.2		4520	4554	2980	3848	1823	2090	1740	700	2669	1494	170	1199
**Dme**	36.7	37.5	41.2	43.8	60.6		7072	3904	3822	1753	1967	1738	713	2476	1460	178	1141
**Aga**	41.3	41.7	47.4	51.5	72.4	61.3		3973	3926	1833	2107	1793	717	2641	1597	177	1214
**Cel**	34.2	37.4	42.2	45.7	65.1	49.9	58.8		12416	1549	1702	1593	697	2235	1368	171	990
**Cbr**	42	42.9	47.5	54.5	79.7	56.7	68.9	84.4		1611	1836	1561	691	2205	1348	168	1083
**Sce**	42.9	43.7	48.8	49.5	72.4	54.8	63.9	60.3	69		2604	4036	683	1867	1321	177	958
**Ncr**	48.3	47.3	52.8	59.2	83.1	59.2	71.9	67.9	77.9	84.3		2560	648	2182	1388	168	880
**Ago**	48.6	47.4	52.9	59.3	82.9	59.9	72.5	69.5	78.3	93.4	96.4		686	1818	1282	172	893
**Ecu**	42.6	39.5	43.5	55.7	77	55.4	66.8	68.4	76.4	77	91.8	91.3		741	567	143	525
**Ath**	29	28.5	32.2	32.4	40.9	33.3	37	32.9	36.4	36.6	39.3	38.6	33.1		2126	199	1295
**Cme**	48.2	45.4	52.1	59.6	83.2	57.6	71.3	68.5	77.7	78.7	90.4	92	90.8	40.9		198	849
**Gtn**	43.5	30.6	33.3	64.9	84.7	54.5	66.7	71.9	77.4	62.7	92.9	88.4	97.2	25.1	91.9		190
**Pfa**	46.2	42.1	46.9	58.7	80.9	57.9	71.6	71.4	78	79.6	91.6	91.3	91.8	36.6	91.2	94.7	

^a^Three-letter abbreviations listed in Methods.

**Table 2:** Bayesian divergence time estimates (± one standard deviation) for eukaryote genome datasets.

**Divergence**	**15 Genomes (5094 aa)^a^**	**12 Genomes (23,571 aa)**	**7 Genomes (132,190 aa)**	**4 Genomes (287,000 aa)**
*Mus – Rattus*	37 ± 5	50 ± 8	40 ± 9	n/a^c^
Primate – Rodent	93 ± 10	117 ± 15	120 ± 20	n/a
Tetrapod – Fish^b^	459 ± 20	460 ± 20	460 ± 20	458 ± 20
Vertebrate – *Ciona*	771 ± 47	756 ± 58	n/a	n/a
*Drosophila – Anopheles*	445 ± 38	500 ± 48	477 ± 51	n/a
Chordate – Arthropod	949 ± 66	912 ± 89	955 ± 92	1109 ± 103
*C. elegans – C. briggsae*	89 ± 13	114 ± 22	n/a	n/a
Coelomata – Nematoda	1166 ± 89	1074 ± 116	n/a	n/a
*Ashbya – Saccharomyces*	311 ± 39	n/a	n/a	n/a
Saccharomycetes – *Neurospora*	900 ± 80	851 ± 96	n/a	n/a
Animal – Fungi	1493 ± 125	1303 ± 155	n/a	n/a
*Arabidopsis – C. merolae*	1414 ± 121	n/a	n/a	n/a
Animal/Fungi – Plant	1671 ± 145	n/a	n/a	n/a

^a^Number of amino acids (aa). ^b^Tetrapod – actinopterygian fish divergence constrained between 425 and 495 Ma [78]. ^c^Divergence not available (n/a) for timing.
