# Supplementary material for: Evolutionary sequence analysis of complete eukaryote genomes
Source: BMC Bioinformatics. 2005 Mar 11;6:53. doi: 10.1186/1471-2105-6-53 (PMC1274250; doi:10.1186/1471-2105-6-53)
Supplement: Additional File 1 — Consensus trees of individual gene trees. Consensus trees of individual gene (panortholog) trees, showing percentage of individual gene trees supporting each node. [file 1471-2105-6-53-S1.pdf]

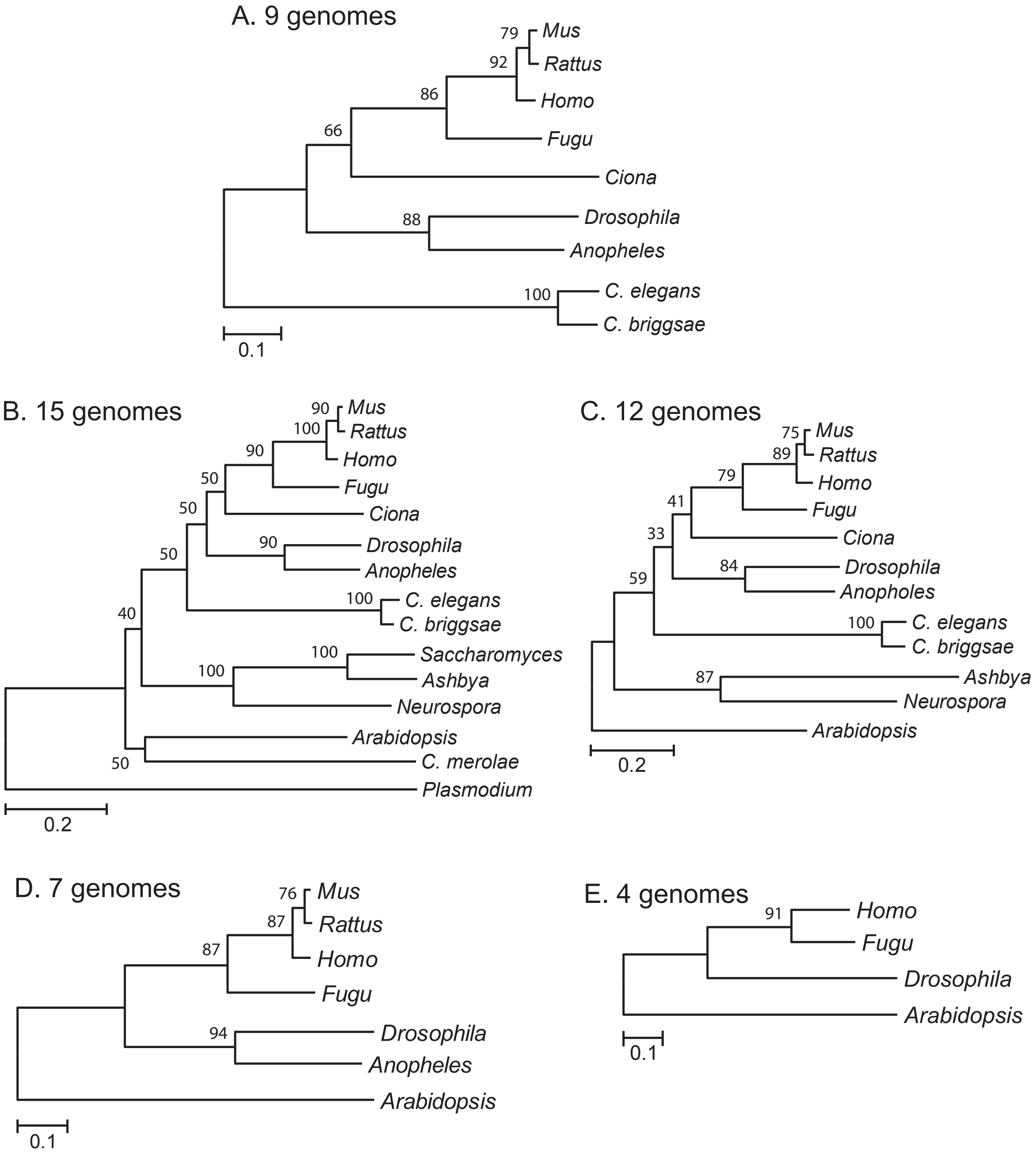

Additional file: Figure 5. Consensus trees of individual gene (panortholog) trees, showing percentage of individual gene trees supporting each node.
